# Large Endometrioma That Triggered a Hypertensive Emergency: A Case Report

**DOI:** 10.1155/2024/7869172

**Published:** 2024-10-23

**Authors:** Varnita Vishwanath, Gregory Marchand, Ali Azadi

**Affiliations:** ^1^Department of Emergency Medicine, Nassau University Medical Center, Long Island, New York, USA; ^2^Department of Gynecological Surgery, Marchand Institute for Minimally Invasive Surgery, Mesa, Arizona, USA; ^3^Department of Obstetrics & Gynecology, University of Arizona College of Medicine, Phoenix, Arizona, USA; ^4^Department of Obstetrics & Gynecology, Creighton University, School of Medicine, Phoenix, Arizona, USA

## Abstract

Endometriosis is a common gynecological condition in women of reproductive age and has variable symptomology such as pelvic pain, menorrhagia, dysmenorrhea, dyspareunia, and infertility. Endometriomas are a form of endometriosis and are characterized by cystic masses most commonly found on the ovaries. This case discusses the management of a rare occurrence of a 25-cm endometrioma in a patient without a prior diagnosis of endometriosis, who presented to the emergency room in an acute hypertensive emergency. It is believed that the large cyst caused a mass effect against renal vasculature precipitating renovascular hypertension that required immediate intervention. This case was approached with minimally invasive surgical removal of the cyst and lysis of adhesions without postoperative complications.

## 1. Introduction

Endometriosis is an estrogen-dependent chronic inflammatory disease characterized by the presence of endometrial glands and stroma outside the uterine cavity [1]. It affects approximately 10% of reproductive-age women worldwide [2]. The prevalence of endometriosis ranges with its broad scope of presentation including 7% in asymptomatic women, 50% in those presenting with infertility, and 70% in those with chronic pelvic pain [3]. Women can develop endometriosis at any age with a peak disease incidence between ages 25 and 45 [4, 5]. Endometriosis classically presents in three different forms including superficial peritoneal implants, deep infiltrating endometriosis, and ovarian endometriomas [6].

Ovarian endometriomas, also referred to as “chocolate cysts,” are cystic masses formed by hemorrhagic endometrial tissue surrounded by ovarian parenchyma [1]. They are the most common form of endometriosis and reportedly occur in 17%–44% of endometriosis patients [7]. While endometriomas most classically occur on the ovary, they may also be found in other regions of the abdomen and pelvis including the bowel or distant sites like the diaphragm. They commonly present as benign and small cysts, and those ≥ 6 cm are encountered less frequently [8]. Their presence may indicate a more severe stage of endometriosis. The definitive technique to diagnose endometriomas is a surgical approach for direct visualization and tissue sampling with histopathology consistent with endometrial glands and stroma [1]. In this paper, we report a rare case of a 25-cm endometrioma in a young woman who presented in a hypertensive emergency likely secondary to poor renal perfusion. This case report was exempted from Institutional Review Board (IRB) approval.

## 2. Case Presentation

A 20-year-old female without a significant past medical or surgical history presented to the emergency department complaining of diffuse intermittent abdominal pain. Upon presentation, a distended abdomen with palpable firmness, moderate tenderness, and guarding was noted. Her blood pressure on admission was 190/121, but her remaining vitals were stable. She presented with acute kidney injury (AKI) reflected by a creatine (Cr) of 2.4 mg/dL and glomerular filtration rate (GFR) of 38 mL/min/1.73 m^2^. To control the patient's hypertensive emergency, administration of intravenous hydralazine was required since the patient remained hypertensive after adequate pain control. At the time of presentation, the patient was nulligravid with her last menstrual period 1 week prior to this hospitalization. Her menstrual periods were regular, occurring every 28–30 days, lasting 4–6 days with moderate flow and mild dysmenorrhea defined as a 2 out of 10 on the pain scale. She endorsed a history of chronic constipation and bloating for several months. She denied menorrhagia, dyspareunia, or chronic pelvic pain.

**Figure 1:**
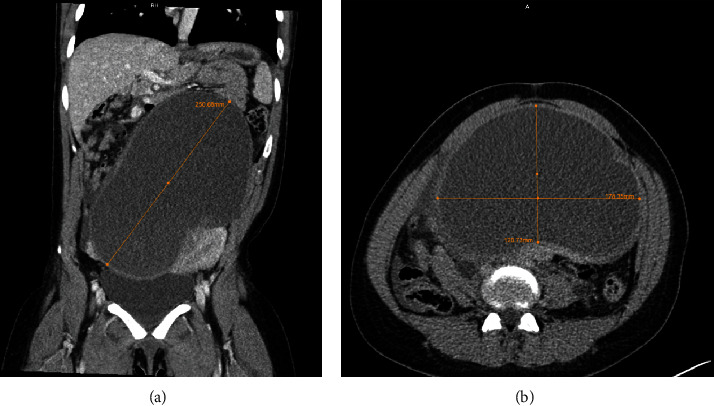
Preoperative contrast-enhanced CT revealed a large unilocular complex adnexal mass in the coronal (a) and axial (b) views, producing a mass effect on the surrounding structures.

**Figure 2:**
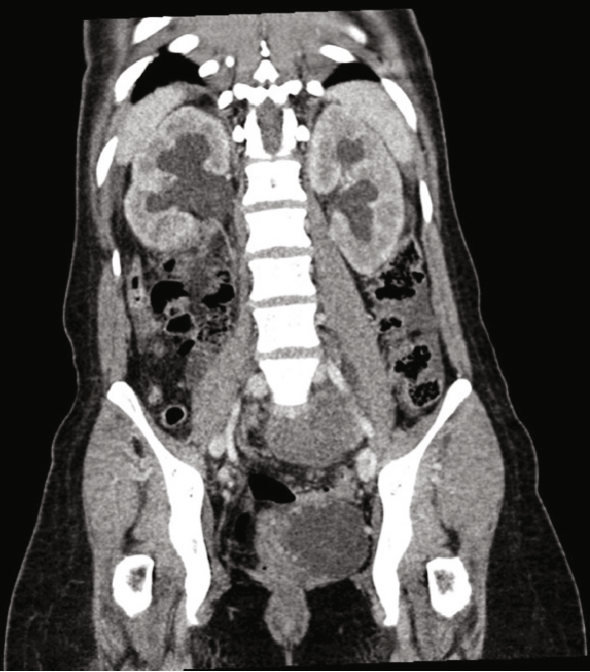
Preoperative contrast-enhanced CT demonstrated bilateral hydronephrosis, secondary to compression from the large adnexal mass.

Given the acute and severe nature of her symptoms, a contrast-enhanced multiplanar computed tomography (CT) scan was performed directly to ascertain the cause of her symptoms. The CT demonstrated a thick-walled complex left adnexal cystic mass measuring 12 × 18 × 25 cm (anterior-posterior by transverse by craniocaudal) (Figures 1(a) and 1(b)), with resultant mass effect and bilateral hydroureteronephrosis (Figure 2). Bilateral nephrostomy tubes were subsequently placed to improve renal function prior to the surgical intervention of the pelvic mass. Of note, the tumor markers including cancer antigen 125, carcinoembryonic antigen, alpha-fetoprotein, and lactate dehydrogenase were negative.

The patient was taken to the operating room for da Vinci–assisted removal of the pelvic mass. Entry to the abdomen was obtained with a 2-cm midline open incision at the umbilicus carried through to the underlying fascia. The cyst wall was noted and a square suture of Vicryl was placed. Using a Bovie, the cavity of the cyst was entered. Drainage from the cyst consisted of dark brown fluid which was extracted with suction. The cyst had decompressed, and the suture was tied. Subsequently, trocars were inserted and the robot was docked.

Under direct visualization, the cystic mass appeared to have originated from the left adnexa with severe adhesions to the pelvic sidewall, sigmoid colon, and the posterior surface of the uterus and cervix. The large size of the endometrioma had caused significant anatomical distortion, making it extremely challenging to identify the ovary and fallopian tube separately from the mass. Therefore, to ensure complete removal of the mass and to prevent potential complications, a salpingo-oophorectomy was performed. For a successful dissection of the mass and salpingo-oophorectomy, ureterolysis was performed. The mass was carefully dissected and the left infundibulopelvic ligament was clamped and cut using the vessel sealer. Multiple endometriotic lesions were noted in the anterior and posterior cul-de-sacs.

The intraoperative and postoperative courses were uncomplicated with normalization of blood pressure and resolution of the AKI. The pathology report confirmed the diagnosis of endometriosis. Prior to the removal of nephrostomy tubes, a nephrostogram was performed which demonstrated appropriate patency of the ureters bilaterally.

## 3. Discussion

Endometriosis is a common gynecological condition among women of reproductive age. A multidisciplinary approach to the management of endometriosis is often warranted as the pathology can affect organs outside of the reproductive system. Ovarian endometriomas make up nearly 35% of all benign ovarian cysts and are the most common form of endometriosis [8]. This case was a rare occurrence of a 25-cm endometrioma in a patient who presented with acute intermittent abdominal pain in a hypertensive emergency. She lacked the classic symptoms associated with endometriosis, such as dyspareunia, dysmenorrhea, or menorrhagia, and had not previously been diagnosed with endometriosis prior to this hospitalization [9]. The aberrant intensity of her abdominal pain in conjunction with severe hypertension necessitated prompt workup and intervention.

While there is a documented association between hypertension and endometriosis in the setting of a chronic inflammatory state [10], the patient in this case presented in a hypertensive emergency which is rare in endometriosis [9]. The proposed mechanism of hypertension, in this case, may be multifactorial in the setting of severe pain and the mass effect of the cyst on the renal arteries. It is well understood that the body's response to acute pain is driven by the sympathetic nervous system which releases catecholamines that raise systemic blood pressure. However, the patient's blood pressure was severely elevated despite adequate pain control. The mass effect of the endometrioma on adjacent vasculature combined with AKI in this patient is believed to have caused renovascular hypertension due to the obstruction of renal arterial blood flow. Low perfusion to the kidney prompts adaptive changes including the activation of the renin-angiotensin-aldosterone system (RAAS) [11]. The release of renin stimulates a cascade of events that activates angiotensin II, which is responsible for systemic vasoconstriction, and releases aldosterone, which retains sodium and water and causes secondary hypertension. Obstructive uropathy secondary to constricted ureters is evident in this patient with bilateral hydroureteronephrosis and also may account for the activation of RAAS [12].

To our knowledge, this is the first documented case of a large endometrioma without a previously known history of endometriosis that presented in an acute hypertensive emergency. The clinical presentation of endometriosis is highly variable. While ovarian endometriomas are a common form of endometriosis, they are typically found to be less than 6 cm in size [8]. Larger endometriomas occupying the abdominal cavity as seen in this case report have been reported less frequently and with a variety of nonspecific presentations. Much like the mass effect noted in this case, documented mass effects secondary to a large endometrioma include large bowel obstruction causing nausea/vomiting [13] and cranial displacement of bowel causing dyspnea [14]. Although rare, documented cases of large endometriomas occupying the abdominal cavity in post-menopausal women have also been reported [6, 7]. This signifies the atypical and variable presentations of endometriosis that should be considered in cases of abdominal complaints in women of not only reproductive age but also postmenopause.

The diagnostic workup of endometriosis includes imaging with ultrasound or magnetic resonance imaging (MRI). MRI is particularly recommended if there is suspected involvement of organs outside the pelvis and for the detection of deep infiltrating endometriosis [15]. Given the severe and acute nature of the case, an MRI was not performed and the patient was directly transferred for surgery. We managed this case with a minimally invasive approach. Excision of the cyst is often recommended as it is associated with improvement in pelvic pain and infertility [9]. Furthermore, our multidisciplinary approach improved the quality of care and outcomes. On 1-year follow-up, this patient remained asymptomatic from endometriosis and was able to conceive.

## Data Availability

The data used for this case presentation is available in medical records at Banner Health System.
